# Construction and Potential Applications of Biosensors for Proteins in Clinical Laboratory Diagnosis

**DOI:** 10.3390/s17122805

**Published:** 2017-12-04

**Authors:** Xuan Liu, Hui Jiang

**Affiliations:** 1 Department of Clinical Laboratory, The Second Affiliated Hospital of Southeast University, Nanjing 210003, China; silentsign@163.com; 2 School of Biological Science and Medical Engineering, Southeast University, Nanjing 210096, China

**Keywords:** immunoassay, biosensors, protein biomarker, serodiagnosis

## Abstract

Biosensors for proteins have shown attractive advantages compared to traditional techniques in clinical laboratory diagnosis. In virtue of modern fabrication modes and detection techniques, various immunosensing platforms have been reported on basis of the specific recognition between antigen-antibody pairs. In addition to profit from the development of nanotechnology and molecular biology, diverse fabrication and signal amplification strategies have been designed for detection of protein antigens, which has led to great achievements in fast quantitative and simultaneous testing with extremely high sensitivity and specificity. Besides antigens, determination of antibodies also possesses great significance for clinical laboratory diagnosis. In this review, we will categorize recent immunosensors for proteins by different detection techniques. The basic conception of detection techniques, sensing mechanisms, and the relevant signal amplification strategies are introduced. Since antibodies and antigens have an equal position to each other in immunosensing, all biosensing strategies for antigens can be extended to antibodies under appropriate optimizations. Biosensors for antibodies are summarized, focusing on potential applications in clinical laboratory diagnosis, such as a series of biomarkers for infectious diseases and autoimmune diseases, and an evaluation of vaccine immunity. The excellent performances of these biosensors provide a prospective space for future antibody-detection-based disease serodiagnosis.

## 1. Introduction

The laboratory immunoassay is an extension of immunological techniques for laboratory diagnosis. It was developed as a consequence of the discovery of various immune substances. In 1894, J. Bordet, a Belgian serologist reported the discovery of alexin [1], which tremendously supported the development of humoral immunity. Based on an in vitro antigen–antibody reaction, serology further formed and developed, and gradually became mainstream in immunology development in the following decades. Basically, according to the various immunological mechanisms and techniques, targets of laboratory immunoassay can be categorized into two groups: (1) immune substances, such as active cells, antigens, antibodies, alexins, cytokines, and cell adhesion molecules; (2) trace substances, such as hormones, enzymes, proteins, and cyclic drugs. In clinical laboratories, qualitative and quantitative determinations of these targets are carried out using proper analytical methodologies, providing significant references for clinical diagnosis. Serodiagnosis, as a noninvasive diagnosis path, compared to tissue biopsy or radiology diagnosis, has shown a competitive status for clinical precaution, especially for early diagnosis, recurrence, and metastasis of tumor, the real-time evaluation of disease development, and curative effects.

As known, antibody expression in human blood can be considered as an indicator of disease generation, development and recovery [2,3,4]. In some circumstances, serum antibodies show better stability and higher concentrations in early stages than the relevant original virus, antigens, and cells [5] and thus could be considered as more efficient biomarkers for disease diagnosis and real-time monitoring. The determination of a specific antibody or combination of several types of antibodies could provide strong evidences of disease existence. Up to now, serodiagnosis has always been the easiest, the most effective, and the most popular analysis method for HIV infection [6]. Between 1968 and 1972, the World Health Organization (WHO) and the International Federation of Immunological Societies (IUIS) decided that a series of globulins, which possessed activity or chemical structures similar to antibodies, were collectively defined as immunoglobulin (Ig). For some infectious diseases, IgM shows an acute increase in early stages of infection, usually around 1 week, and can be used as a biomarker for early laboratory diagnosis [7,8]. The specific IgG shows an increase about four weeks after infection and lasts for the long term, years sometimes, and could be used as a biomarker for the evaluation of recovery [9,10]. In addition, the Igs are always involved in hypersensitivity of the body [11,12]. For example, IgE can induce type I hypersensitivity, and specific IgEs are detected for the determination of allergens. Autoimmune diseases (AIDs) are a series of diseases that occur as a result of the loss of immunologic tolerance to self-antigens, showing various cases with common characteristics [4]. Each AID has a characteristic antibody spectrum that can be objectively evaluated as an indicator for disease prediction, early diagnosis, and prognosis [13]. Furthermore, antibody immunoassays based on various techniques have also been widely used for vaccine immunity evaluation in clinical laboratories [14]. Thus, serological antibody analysis has shown great significance for clinical laboratory diagnosis.

According to the biological properties of targets, several methodologies have been used in laboratory immunoassays [15,16,17], such as precipitation-reaction-based assays, radio-immunoassays, fluorescent immunoassays, enzyme-based immunoassays, and chemiluminescent (CL) immunoassays. Especially, enzyme-linked immunosorbent assay (ELISA)-based methods are the most widely used in clinical laboratories [18]. Although these heterogeneous-reaction-based methods have been providing acceptable support for clinical diagnosis, antibody/antigen determination still requires novel methodologies that are more applicable for early molecular event detection with better sensitivity and easier operation [19]. Biosensors are bioanalytical devices integrating biorecognition elements and suitable transducers, which could be further designed with various signal amplification strategies to obtain extremely high sensitivity [2]. With plenty of advantages, such as strong specificity to targets, rapid analysis, high accuracy, easy operation, and low cost, biosensors have expansive applications for diverse targets, including cyclic drugs [20], proteins [21,22,23], nucleic acids [24,25,26,27], and cyclic tumor cells [28,29,30]. Immunosensors have played a crucial role in the determination of tumor markers [31,32,33], significantly improving early diagnosis and prognosis of various cancers. Molecular identification unit is the basis of specific determination, which specifically recognizes and captures the target, and then induces a series of physical or chemical changes. These changes could be converted to detectable optical or electrical signals by the transducers. Modern electrical and optical techniques have shown unique properties for the construction of biosensing platform. With a combination of diverse nanomaterials, more and more novel biosensors have shown excellent properties, which can well meet the demands of clinical diagnosis. Moreover, the portability of biosensors even further enables the development of point-of-care (POC) devices [34], which is proficient for home use, as well as the continuous monitoring of treatment efficiency. 

In this review, we will focus on the development of biosensors for protein detection, including fabrication procedures, combination of signal amplification, utilization of various detection techniques and methodologies (Section 2). Protein biosensing platforms are always universal for all kinds of proteins based on proper optimization. Antibody biosensors with potential applicability for clinical diagnosis for the detection of biomarkers of infectious diseases and AIDs and for the evaluation of vaccine immunity are then summarized (Section 3). In the conclusion section, the development of next-generation biosensors for serodiagnosis is briefly discussed. 

## 2. Biosensors for Protein Immunoassays

To further improve the performances of biosensors for proteins, great efforts have been made in fabrication procedures, supporting materials, signal amplification strategies, detection techniques, and so on. In this section, typical techniques and methodologies for protein biosensing are summarized. These strategies may be applicable for antibody detection in the following section.

### 2.1. Fabrication of Immunosensors

**Figure 1:**
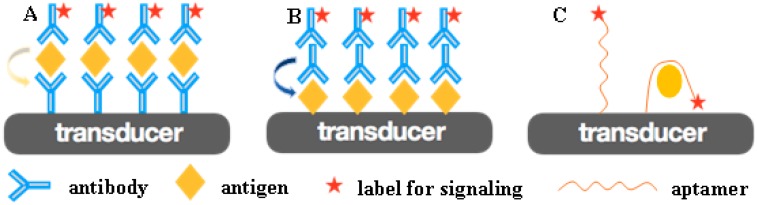
(**A**) The antibody works as an MRE to recognize and capture the specific antigen in sandwich-mode immunosensing. (**B**) Antigen works as an MRE to recognize and capture the specific antibody in sandwich-mode immunosensing. (**C**) Aptamer works as an MRE to recognize and capture the specific protein.

In order to develop a highly sensitive and specific immunosensor, a molecular recognition element (MRE) with a high affinity must firstly be immobilized onto the sensing interface. Specific antibodies/antigens [33,35] and aptamers [36] are two kinds of well-established MREs (Figure 1). The antigen–antibody reaction is the basis of immunoanalysis in clinical laboratories, and modern immuno-labeling techniques using highly sensitive substances as labels have further broadened the scope of immunological techniques. In recent years, oligonucleotide aptamers have exhibited high affinity and specificity, as well as high speed in selection, synthesis, and scale-up, which has attracted great attention in terms of diagnosis and therapeutics [37,38,39]. These specific MREs are firstly immobilized on the transducer’s surface as capture probes, and the target proteins can then be specifically recognized by capture probes, linking to the interface of biosensors. The recognition reaction may be accelerated by external driving forces, such as stirring and electricity [40]. The transducer converts the biological response generated by the interaction of specific recognition pairs into a measurable signal to the receptor [41].

**Figure 2:**
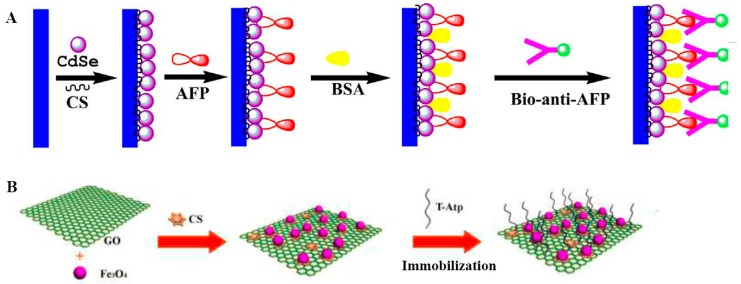
(**A**) Quantum dots (QDs) acted as biocompatible matrices for the immobilization of alpha fetoprotein (AFP) and photoactive materials to increase the photoelectrochemical (PEC) signals. (**B**) Chitosan-modified magnetic oxide graphene composite (CS@Fe_3_O_4_@GO) worked as supporting matrices for thrombin aptamer. Reproduced from [37,42] with permission from Elsevier.

Generally, to further increase the stability and reaction efficiency of biosensors, biocompatible matrices are needed to immobilize capture probes and to provide the circumstances appropriate for heterogeneous biological reactions [37,42]. Many nanomaterials have been used for the construction of biocompatible matrices, and the matrices could also act as electron transfer media or tracing signal emitters (Figure 2).

### 2.2. Electrochemical (EC) Immunosensors

An efficient EC transducer matrix for a biosensing device requires specific characteristics, such as fast electron transfer, high stability and surface area, good biocompatibility, and the presence of specific functional groups, to facilitate biomolecule attachment. EC immunosensors have dramatically developed in recent decades, showing high sensitivity, ease of operation and a low cost, and only requiring a small dose of analytes [34]. Nanomaterial-based EC immunosensors have shown extensive advantages, leading to a greater loading amount of MREs, a faster electron transfer speed, and a more convenient combination of signal amplification strategies [2]. For example, Au nanoparticles (NPs) [43,44], carbon nanotubes (CNTs) [21], and graphene [36] have been widely used to preserve the activity of biomolecules and to enhance the electron transfer between redox species, increasing sensitivity. In addition, modern EC immunosensors exhibit an excellent detection performance for whole blood tests in a fast and simple way [31,45], with remarkable sensitivity and selectivity, offering EC biosensors with a benefit for transition toward POC application [34].

**Figure 3:**
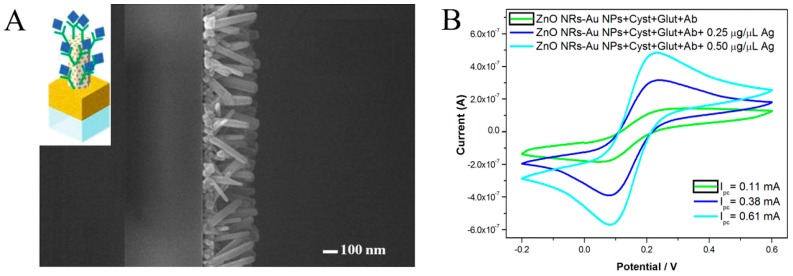
(**A**) SEM surface micrographs of ZnONR–AuNP nanohybrids from top view. Inset: Schematic model of a ZnONR–AuNP nanohybrid immunosensor for CA125 detection. (**B**) Cyclic voltammograms of ZnONR–AuNP-hybrid-modified electrodes immobilized with cystamine (Cyst), glutaraldehyde (Glut), Ab, and 0.25 or 0.50 μg μL^−1^ of CA125 antigen. Reproduced from [46] with permission from Elsevier.

Plenty of EC immunosensors have been constructed for early diagnosis with different EC techniques. A cyclic voltammogram (CV)-based immunosensor using directly grown ZnO nanorod (NR)–AuNP nanohybrids as supporting material for immobilizing antibodies as capture probes (Figure 3) was developed for the specific detection of ovarian cancer antigen CA125, showing a limit of detection (LOD) of 2.5 ng μL^−1^ under CV measurements [46]. These in-situ grown nanohybrids provided an efficient means of immobilizing capture probes. Although the LOD was not extremely high compared with other EC biosensors, these results were sufficient for the potential application for clinical diagnosis with simple operation. Recently, viologen-single-walled CNTs (SWCNTs) hybrids were synthesized for an enrichment in antibodies and enzymes and for an increase in electron transfer between transducer and electroactive materials. Based on the dual amplification efficiencies, only a trace sample was needed, and dramatically enhanced sensitivity for TGF-β1 cytokine detection was achieved, with a linear range of 2.5~1000 pg mL^−1^ and an LOD of 0.95 pg mL^−1^ [21]. An immunosensor for POC enterovirus 71 detection was proposed for both colorimetric and EC measurements, which was proved to possess good performance for early diagnosis and control of the related epidemics [22]. Using an AuNP-coated indium tin oxide (ITO) electrode as the substrate, a concentration of 1.0 ng mL^−1^ could be read directly with the naked eye, enabling POC application for virus detection. On the other hand, CV-based determination in the same detection cell showed an LOD of 0.01 ng mL^−1^. Although a lower LOD was found via CV measurement, colorimetric measurements that have an acceptable resolution are more beneficial for potential POC applications. Besides CV, other electric techniques such as differential pulse voltammetry (DPV) [47] and square wave voltammetry (SWV) [48] have also been widely used for protein immunosensing.

**Figure 4:**
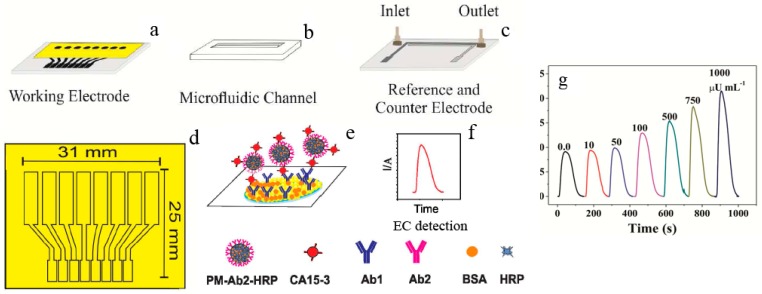
Construction of a disposable microfluidic immunoarray device: (**a**) working electrode; (**b**) microfluidic channel and (**c**) reference and counter electrodes; (**d**) pattern and size of the working electrode array; (**e**) the formation of the immunosandwich structure; (**f**) EC detection strategy; (**g**) amperometric responses of CA15–3 standard solutions at −0.2 V vs. Ag/AgCl after the injection of a mixture of H_2_O_2_ and HQ. Reproduced from [23] with permission from the American Chemical Society.

In clinical diagnosis, a small sample volume, a high sensitivity, and multi-channels for simultaneous tests meet the potential requirements precisely. A disposable microfluidic immunoarray device was designed for the rapid and low-cost detection of CA15-3 (Figure 4), a biomarker for breast cancer, based on a double-sided adhesive card with a microfluidic channel and a screen-printed array with 8 electrodes, as well as an inexpensive home cutter printer and some low-cost materials. This device showed an LOD of 6 μU mL^−1^, requiring as little as 2 μL of serum samples and allowing 8-channel simultaneous detections [23]. 

Besides antibodies/antigens, aptamers have also been widely used as capture probes for the construction of immunosensors, named aptasensors, with good selectivity and stability. For example, based on bridged rebar graphene, a novel label-free aptasensor was built for pathogenic bacteria *E. coli* O78:K80:H11 detection using a screen-printed electrode and an impedance detection technique [36]. The specific anti-*E. coli* DNA aptamer with K_d_~14 nM was immobilized on a bridged-rebar-graphene-nanostructured electrode and used as a capture probe, showing an LOD as low as ~10 cfu mL^−1^. 

To enhance detection sensitivity, various signal amplification strategies have been combined with biosensing platforms [2,21,32,33,43,44]. For example, a dual signal amplification strategy was employed in fabricating ultrasensitive EC immunosensor for alpha fetoprotein (AFP) detection [32]. A ZnONR/AuNP-hybridized reduced graphene nanosheet was utilized to significantly increase the loading capacities of Ab1. Then, a horseradish-peroxidase (HRP) bioconjugated detection antibody (Ab2)-functionalized Au@ZnO composite increased the amount of peroxidase-like catalytic activity, thus largely enhancing the response signals.

### 2.3. Photoluminescent (PL) Immunosensors

**Figure 5:**
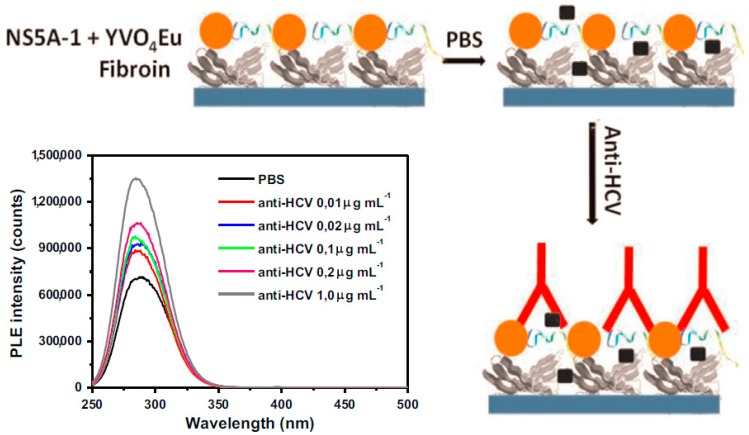
Schematic representation and PL spectra of the as-prepared biosensor. The LbL film of SF/NS5A-1+YVO_4_:Eu^3+^ NPs showed specific recognition in the presence of anti-HCV antibodies. PL spectra obtained for the LbL film containing five bilayers of fibroin/NS5A-1+YVO_4_:Eu_3_ NPs in the presence of different concentrations of anti-HCV. Reproduced from [33] with permission from Elsevier.

The analytical performance of PL immunosensors, similar to that of EC biosensing, has been largely improved by a combination of nano-techniques [33,35,49,50]. Lima et al. [33] reported a sensing platform for an anti-hepatitis C virus (HCV) antibody using YVO_4_:Eu^3+^ luminescent NPs as a PL probe (Figure 5). The peptide NS5A-1 derived from HCV NS5A protein together with the NPs was immobilized layer by layer (LbL) onto silk fibroin, by which the anti-HCV antibody was detected in the range of 0~0.01 μg mL^−1^. The sensing platform showed advantages of easy operation and high sensitivity. However, the platform lacked a discussion of sensing mechanism and selectivity, which limited the extension application of the immunosensor. Bhatnagar et al. [35] designed a “switch off” Förster resonance energy transfer (FRET)-based biosensor using graphene quantum dots (GQDs) as a donor for early detection of heart attacks. In the presence of the target cardiac Troponin I (cTnI) antigen, the FRET path was blocked, and the PL signal of the GQDs then recovered with the increase in target concentration, showing a linear response to cTnI from 0.001 to 1000 ng mL^−1^. The rapid detection was also well catered to the clinical detection requirements of the target cTnI. 

With the assistance of various nanostructures, PL immunosensors with luminophores immobilized on proper substrates showed a much higher detection sensitivity than traditional detection systems in aqueous solutions. For example, a rapid, sensitive, and low-cost PL immunosensor for the determination of aflatoxin B1 (AFB1) was developed [49] based on porous silicon covered by a thin layer of gold, where anti-AFB1 acted as an MRE. The crystalline AuNPs uniformly coated the surface of the porous silicon pores to form an Si/Au structure. The immunosensor was tested in a wide range of AFB1 concentrations, from 0.001 to 100 ng mL^−1^. A PL biosensor for grapevine virus A-type proteins (GVA-antigens) was developed based on ZnO thin films deposited via atomic layer deposition (ALD) [50]. The GVA-antigen detection was performed via an evaluation of the changes and behavior of the corresponding luminescence band. The sensitivity of the as-formed label-free biosensor showed a linear range from 1 pg mL^−1^ to 10 ng mL^−1^.

### 2.4. Photoelectrochemical (PEC) Immunosensors

PEC biosensing couples photoirradiation and EC detection techniques together and benefits from simple operation, inexpensive equipment, and good portability, providing a novel analytical strategy for protein analysis and attracting worldwide attention [51]. Owing to the different energy forms of the excitation source and readout signal, the PEC method, compared with traditional EC methods, shows a lower background and a higher sensitivity [52]. Nanostructures usually possess preferable thermal and chemical stabilities and have been widely used as photoanodes in PEC sensors [52]. In order to determine comprehensive design guidelines for more advanced PEC sensors, the previous review categorized recent PEC biosensor examples into three signaling principles [53], i.e., reactant determinants, electron transfer, and energy transfer. To further increase the photocurrent, many nanomaterials and nanostructures were incorporated into the construction of PEC biosensors to accelerate charge transfer [38,51,54,55,56] and increase the accessible surface [57].

**Figure 6:**
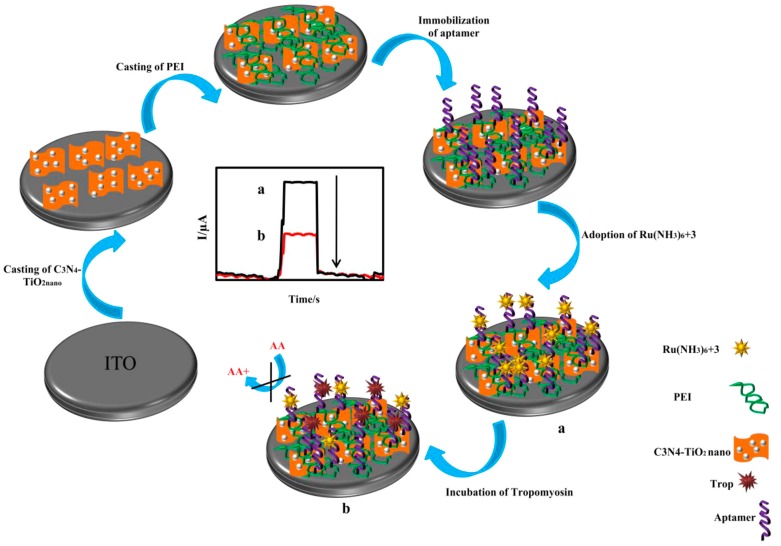
The schematic illustration for fabrication and detection procedures of a PEC aptasensor. Inset: Time-based photocurrent response curves in the absence (**a**) and presence (**b**) of tropomyosin. Reproduced from [38] with permission from Elsevier.

Semiconductors have been widely used in PEC immunosensors, exhibiting a high detection sensitivity and an LOD at the pg mL^−1^ level when combined with proper signal amplification strategies. Zhang et al. [51] reported a PEC immunosensor based on Mn-doped CdS (CdS:Mn QDs) on graphitic carbon nitride (g-C_3_N_4_) nanosheets as a photoactive material for the sensitive detection of a prostate-specific antigen. The signal was amplified through DNAzyme concatamers on AuNPs accompanying enzymatic biocatalytic precipitation, showing an LOD as low as 3.8 pg mL^−1^. These nanohybrids highly improved sensing sensitivity and showed results comparable to those attained via ELISA methods. A visible-light-driven PEC method for the detection of shrimp allergen tropomyosin was constructed using g-C_3_N_4_ and TiO_2_ as photoactive nanomaterials [38]. Ascorbic acid worked as an electron donor, and Ru(NH_3_)_6_^3+^ was adsorbed on the specific aptamer to enhance the photocurrent signal (Figure 6). After recognition between tropomyosin and the capture aptamers was established, the absorbed Ru(NH_3_)_6_^3+^ were released from aptamers and prevented the electron donor from scavenging photogenerated holes to the photoactive-material-modified electrode, based on which the quantitative detection of the target obtained a concentration range of 1~400 ng mL^−1^, with an LOD of 0.23 ng mL^−1^. This sensing process could be performed in the absence of antibodies and enzymes, overcoming the drawbacks of the clinically used ELISA method for tropomyosin detection. A *p*-type semiconductor, *p*-CuBi_2_O_4_, was used as a photocathode with hemin as the photocurrent enhancer to design a split-type PEC immunosensor for AFP detection [54], where AuNPs were immobilized on the fluorine-doped tin oxide electrode as a front contact of *p*-CuBi_2_O_4_ to enhance the efficiency of charge separation. The hemin-based G-quadruplex was labeled on the AuNP and acted as the signal probe, showing a wide linear dynamic range from 50 pg mL^−1^ to 20 ng mL^−1^, with an LOD of 14.7 pg mL^−1^. Neto et al. [55] reported a PEC platform for the immunodiagnosis of canine leishmaniasis using two kinds of peptides from two different proteins, and high specificity and selectivity toward the recognition of *L. infantum* antibodies were demonstrated. The sensing platform was firstly constructed by a double-layer electrodeposition of ZnO and CdS QDs, providing a more sensitized photocurrent response. The immunosensor was able to discriminate between positive and negative canine serum samples at low cost. Recently, a PEC immunosensor based on CdAgTe QDs and dodecahedral AuNPs, stabilized by ionic liquid, was fabricated for the specific detection of cTnI [56]. Under the enhancement of AuNPs, the photocurrent showed a more than 10-fold amplification. The relative photocurrent variation upon the formation of the antibody–antigen complex was used for quantitative detection. The PEC immunosensor showed a relative photocurrent variation directly proportional to the logarithm of cTnI concentration between 5.0 pg mL^−1^ and 20.0 ng mL^−1^, with an LOD of 1.756 pg mL^−1^. Ge et al. [58] proposed an interesting PEC technique using CL as the internal light source for a sandwich immunoassay of CA 125. Hybrids of N-aminobutyl-N-ethylisoluminol, HRP, and CA 125 antibodies were immobilized on graphene oxide and showed excellent CL activity. The ZnONRs grew on a reduced-graphene-oxide-modified paper working electrode with the deposition of CdS QDs, resulting in an enhanced excitation and photo-to-electric conversion efficiency. The immunosensor exhibited a linear range from 5.0 × 10^−4^ to 500 U mL^−1^, with an LOD of 2.0 × 10^−4^ U mL^−1^.

**Figure 7:**
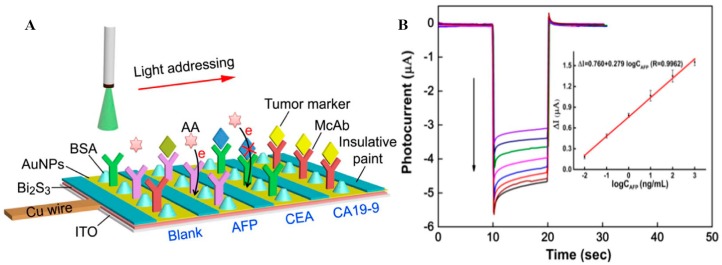
(**A**) Schematic representation for the rapid detection of multiple biomarkers on a single PEC sensor. (**B**) Photocurrent responses and calibration plot (inset) of AFP detection at concentrations of 1000, 100, 10, 1.0, 0.1, 0.01, 0.005, and 0 ng mL^−1^ (from top to bottom). Reproduced from [59] with permission from Elsevier.

As is well known, many cancers show more than one tumor marker overexpressed in the serum of a patient, so the simultaneous and accurate testing of multiple tumor markers may improve the diagnosis of certain types of tumors. A light addressing strategy-based label-free PEC sensing platform was designed for multiple tumor marker detection at the same time on a single electrode [59] (Figure 7). Uniform photovoltaic material Bi_2_S_3_ with a high conversion efficiency in visible light ranges was firstly modified on an ITO electrode by a novel two-step constant potential deposition method. By immobilization of three specific antibodies on the sensor interface, the PEC immunosensor achieved rapid and sensitive simultaneous detection for AFP, carcinoembryonic antigen (CEA), and cancer antigen 19-9 (CA19-9), showing very similar analytical performances, with calibration ranges of 0.01~100 ng mL^−1^ for CEA and AFP and 0.1~1000 U mL^−1^ for CA19-9.

### 2.5. Electrochemiluminescent (ECL) Immunosensors

ECL is a process that converts EC energy to radioactive energy. A coreactant-type ECL process involves the production of reactive intermediates from the reaction between luminophores and appropriate coreactants at the surface of an electrode under a certain potential [60]. The ECL emission is then produced by the excited states obtained from intermediate reaction under a variety of conditions. With the advantages of a lower background signal, a higher sensitivity, and a strong tolerance against interferences, ECL-based biosensors have been widely used for immunoassays in recent years. Based on traditional coreactant–ECL systems of Ru(bpy)_3_^2+^ and its derivatives/ammonium salt [61,62] and luminol/H_2_O_2_ [63], ECL biosensors have been constructed for various targets. Due to their excellent stability, their wide application range of pH, and their EC reversibility, Ru(bpy)_3_^2+^-based ECL immunoassay systems have been widely used in clinical laboratory diagnoses.

Recently, QDs [64,65] have shown competitive ECL properties for biosensing, with approaches of further bio-functionalization and tunable ECL emission potentials tuned by surface microstructures [66]. As the ECL emission from pure QDs, compared to the conventional ECL emitters of Ru(bpy)_3_^2+^ or luminol always show relatively low intensity, some effective signal amplification strategies for QDs based ECL have been selected to overcome this limit and further promote their applications in analytical fields [65,66]. Because of their combination of various QDs [66] or QDs–Ru(bpy)_3_^2+^–luminol ECL systems [67,68,69], sensing platforms for multiple analytes have attracted great attention for potential clinical laboratory diagnoses. Furthermore, immunomagnetic-ECL sensing platforms have also been developed by loading nano-ECL luminophores or recognition elements inside the magnetic porous materials, providing an effective separation and enrichment tool for signal amplification with very easy operation [67,68].

**Figure 8:**
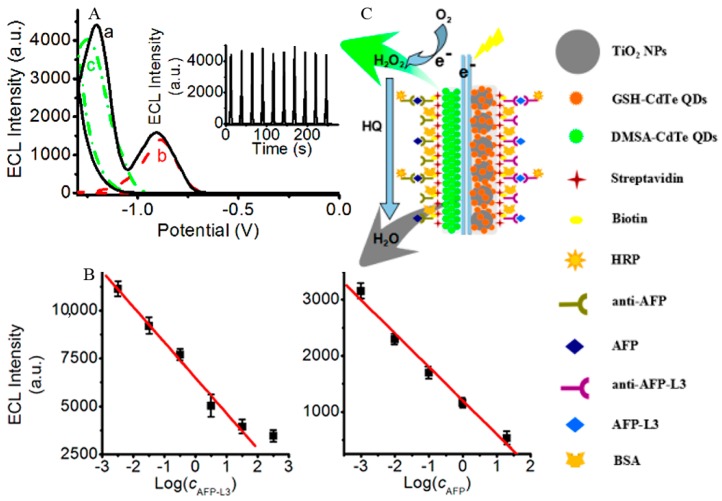
(**A**) ECL curves of simultaneous scan of DMSA-CdTe QDs and TiO_2_-GSH-CdTe QD composites; individual scan of (a) DMSA-CdTe QDs, (b) TiO_2_-GSH-CdTe QD composites, and (c) modified ITO electrodes. Inset: Continuous cyclic simultaneous scans of DMSA-CdTe QDs and TiO_2_-GSH-CdTe QD composites modified ITO electrodes. (**B**) Linear calibration plots for detection of AFP-L3 (left) and AFP (right). (**C**) Sandwich immuno-structure of HRP-labeled antibody–antigen biotinylated antibody on the immunosensor and detection procedures of analytes (green arrow: luminescence; gray arrow: quenching). Reproduced from [66] with permission from the American Chemical Society.

Increasingly, rather than developing new mechanisms or signal amplification strategies for single target analysis, ECL immunosensors have been used for the simultaneous detection of multiple targets. These achievements are more consistent with clinical application requirements, so fundamental research has moved on to practical applications. An ECL potential-resolution dual-target immunosensor was constructed by using two kinds of QDs for the simultaneous detection of AFP and its subtype, AFP-L3. A difference in ECL peak potential of 360 mV was produced via different surface microstructures (Figure 8). The immunosensing was completed in one CV scan, showing a detection linear range of 3.24 pg mL^−1^~32.4 ng mL^−1^ and 1.0 pg mL^−1^~20 ng mL^−1^ for AFP and AFP-L3, with LODs of 3.24 pg mL^−1^ and 1.0 pg mL^−1^, respectively [66]. Babamiril et al. [67] reported a potential-resolution ECL immunoassay for the simultaneous determination of tumor markers AFP and CEA, using QDs and luminol as signal probes. Polyamidoamine dendrimer (PAMAM) and magnetic Fe_3_O_4_–SiO_2_ beads were used as the carrier for immobilizing CdTe@CdS QDs and luminol, forming a dual signal amplification strategy. In the presence of H_2_O_2_, ECL signals were generated at potentials of −1.12 V and +0.6 V (vs. Ag/AgCl), respectively. Both tumor markers could be detected by this method with extremely high sensitivity in the concentration range of 0.25 fg mL^−1^~20 pg L^−1^, with an LOD as low as 0.10 fg mL^−1^. Another multiplex ultrasensitive ECL immunoassay for the simultaneous determination of CA 15-3 and CA 125 was constructed using QDs and Ru(bpy)_3_^2+^ as ECL probes via a wavelength-resolution mechanism [68]. In the presence of tripropylamine as a coreactant, ECL emission occurred at +1.2 V (vs. Ag/AgCl) and could be split into two different wavelengths of 500 and 620 nm, respectively. The immunosensor also used PAMAM and magnetic Fe_3_O_4_–SiO_2_ beads as the carrier to realize dual signal amplifications, showing a wide linear range of 1 μU mL^−1^~1 U mL^−1^ and 0.1 mU mL^−1^~100 U mL^−1^, with very low LODs of 0.1 μU mL^−1^ and 10 μU mL^−1^, respectively. In a recent work, Zhou et al. [69] reported a triple-channel ECL immunosensor for the simultaneous determination of latent tuberculosis infection markers based on three ECL emitters—CdS QDs, carbon QDs, and luminol—integrated together onto AuNPs and magnetic beads. Interferon-gamma (IFN-γ), tumor necrosis factor-alpha (TNF-α), and interleukin (IL)-2 specific antibodies were separately immobilized on three spatially resolved areas of a patterned ITO electrode to capture the corresponding targets, and the ECL intensities reflected the concentrations of IFN-γ, TNF-α, and IL-2 in the concentration range of 1.6~200 pg mL^−1^. 

### 2.6. CL Immunosensors

CL detection does not require external light sources but a simple optical system, which has low background signals and high sensitivity, simple equipment and operation, wide linear range, good reproducibility, and no pollution [70]. Luminol/H_2_O_2_ luminescence is the most widely used system for CL detection [71]. As one of the most sensitive means of detection, CL detection has been widely used for fluidic immunoassay (FIA) [70,71,72], forming a combined technique of CL-FIA that has high sensitivity due to the CL and high selectivity due to the FIA. Moreover, CL detection is also a benefit for the construction of ultrasensitive immunosensors [3,17,37].

A CL-based fiber-optic biosensor was constructed for the detection of crimean-congo hemorrhagic fever (CCHF) IgG antibodies [3] and is 10 times more sensitive than the colorimetric ELISA method. The capture biomolecules were firstly immobilized at the enface tip of an optical fiber and worked via sandwich mode for detection. The fiber-optic immunosensors with a small size and relatively high detection sensitivity present an alternative means for POC applications. The analytical performance was evaluated by the detection of sera from two CCHF patients in Turkey. The detection results indicated that small amounts of antibodies could be detected at early stages of infection, providing a capacity for use as a POC diagnostic system of CCHF. Aptamers also enable good performances in terms of high stability and detection sensitivity. Sun et al. [37] reported an ultrasensitive CL aptasensor prepared for thrombin detection based on iron porphyrin catalyzing luminol/H_2_O_2_ luminescence, showing a linear concentration range of 5.0 × 10^−15^~2.5 × 10^−10^ M, with an LOD of 1.5 × 10^−15^ M. 

Zong et al. [17] developed a CL-array-based disposable immunosensor for the determination of cardiac troponin T (cTnT). AgNPs loaded with guanine-rich DNA sequences and capture antibodies functioned as tracing tags that could catalyze the CL reaction of a luminol-p-iodophenol/H_2_O_2_ system after the formation of a sandwich immunocomplex on the array. The method showed a wide linear range from 0.003 to 270 ng L^−1^, with an LOD down to 84 fg L^−1^ and a throughput as high as 44 tests h^−1^.

### 2.7. Immunosensors Based on Other Techniques

Besides the methods described above, surface plasmon resonance (SPR) [73,74,75,76], microcantilever (MCL) [77,78], and piezoelectric [79,80,81] techniques have also been used for the construction of immunosensors. Subject to the drawbacks of the techniques themselves, such as interference and the low applicable range, immunosensors based on these techniques are not as widely used as those immunosensors mentioned above.

Briefly, SPR is an optical detection technique based on reflection and refraction, showing highly stable biomolecules and high light intensity, which is beneficial for immunosensing. In the SPR sensing system, one side of the chip is modified with capture probes, and the other side contains a thin metal film, usually made of gold. When the solution containing targets flows by, the mass of the molecules bound on the gold film varies, leading to a proportional change in SPR angles. As there are no other procedures such as labeling required for the sensing system, SPR has a significantly easier process than other optical techniques. Magneto-plasmonic NPs have also been employed to enhance the signals of SPR spectroscopy to obtain higher detection sensitivity [76]. 

MCL-based biosensors are also gaining importance for bioanalysis. When targets such as antibodies or antigens bind to the cantilever surface, there will be a change in the resonance frequency and amplitude of the cantilever, which is the basis for biosensing. MCL-based sensors generally operate in two modes: static mode and dynamic mode. The dynamic mode detects the change of resonant frequency caused by mass-loading, with an ultrahigh sensitivity ranging from femtogram to attogram, which is much better than that of the static mode. 

Quartz crystals are the most commonly used piezoelectric transducers in biosensing applications [81]. The produced charges by piezoelectric transducers are in direct proportion to the surface mass variation. The sensitivity of piezoelectric biosensors depends on the oscillating frequency as well as the area of the electrodes on the resonator. Quartz crystals are usually manufactured for frequencies from a few tens of kilohertz to tens of megahertz, which is related to the thickness of the resonator, and are a benefit for the construction of immunosensors as quartz crystals are conducive to label-free, low-cost, and direct detection.

## 3. Potential Applications of Antibody Biosensing for Clinical Laboratory Diagnoses

Due to the advantages obtained from the combination of various nanomaterials, detection techniques, and signal amplification strategies mentioned above, biosensors for antibody detection have shown unique specificities and high sensitivities toward the corresponding analytes. This has allowed for the analysis of highly complex matrices such as blood and other physiological fluids with easy operation and high accuracy.

### 3.1. Biosensors for Markers of Infectious Disease

Because of the WHO’s goal to increase the global capacity to monitor and control the major epidemics and pandemic threats, sensitive, rapid, and predictable detection of specific antibodies by biosensors has become particularly important for clinical serodiagnosis [3,5,6,55,82,83,84,85,86,87,88,89,90].

An electrochemical impedance spectroscopy (EIS)-based biosensor was reported to detect antibodies against plasmodium vivax, a causing agent of malaria [82]. The biosensing procedures for this method of detection can be done within minutes, and only a drop of unmodified blood serum was needed. Using the specific antigen as an MRE and CNTs to enhance the electric properties, electrical changes could be measured at antibody concentration as low as 6~50 pg L^−1^, and as high as ~70 μg L^−1^. Recently, another EIS-based immunosensor was also developed based on CNTs deposits [83]. The bioreceptor unit, biotin-modified cholera toxin B subunit, was immobilized with the nitrilotriacetic acid-Cu(II) complex. After optimization, the resulting EIS cholera sensor showed excellent reproducibility, increased sensitivities, and achieved a satisfying LOD of 10^−13^ g mL^−1^. 

A universal biosensor based on an antibody-catalyzed water oxidation pathway was constructed for the detection of antibodies [84]. The singlet oxygen (^1^O_2_^*^) could be produced by a photosensitizer immobilized on the electrode at the first place. A polymer brush-haptens-modified surface was constructed to recognize specific antibodies with high affinity and specificity. The catalytic activity of these antibodies produced multiple mole equivalents of H_2_O_2_ per antibody, and the antibody quantitative was performed via the SWV-based detection of H_2_O_2_. Although this biosensor was not built specially for the detection of infectious disease biomarkers, as the author mentioned, it could be extended to build biosensors for the detection of all relevant biomarkers of infectious diseases.

Robust biosensors for the detection of infectious disease markers, with a simple setup, low-cost microelectronic circuits, a small volume requirement, and good sensitivity, are listed in Table 1. The achievements of these detection platforms and devices opened new avenues for the early diagnosis of infectious diseases and helped to control major epidemics and pandemic threats.

### 3.2. Biosensors for AIDs Markers

AIDs are characterized by the presence of autoantibodies in the serum of affected patients. Sensitive and accurate biosensing systems for these biomarkers can not only significantly aid in the early diagnosis and clinical management of AIDs but also help to establish therapeutic strategies [4]. Biosensors for the detection of autoantibodies specific to AIDs, including antiphospholipid syndrome (APS) [91,92,93,94], rheumatoid arthritis (RA) [95,96,97,98,99], systemic lupus erythematosus (SLE) [100,101,102,103,104,105,106,107], multiple sclerosis (MS) [108,109,110,111], and celiac disease (CD) [112,113,114,115,116,117,118,119,120,121,122,123,124], are now constructed based on diverse techniques with many advantages, including high sensitivity and easy operation. 

For optical methods, a parallelized, label-free optical biosensor was reported to simultaneously evaluate APS biomarkers in a single measurement. It worked in a serum matrix with a sample volume of 10 μL and was faster than the routinely performed ELISA [91]. A biomimetic optical sensor for APS-specific autoantibody β2-glycoprotein-I detection was constructed with an LOD of 5.62 mg L^−1^ [92]. A highly sensitive magnetic immunosensor was designed for anti-CCP autoantibodies based on SERS detection for the early diagnosis of RA [96], with an LOD as low as 13 pg mL^−1^. 

For electrochemical methods, Villa et al. [95] reported a MWCNT-polystyrene transducer based amperometric biosensor for the diagnosis of RA by detection of serum anti-citrullinated peptide antibodies (ACPAs), which was eventually applied to the detection of ACPAs in human sera. Derkus et al. [111] reported a highly sensitive impedimetric immunosensor for the determination of a MS autoantibody, anti-myelin basic protein antibody, based on CV and EIS detections in short response times, showing LODs of 0.1528 ng mL^−1^ and 0.1495 ng mL^−1^, respectively. This immunosensor also yielded acceptable results for human cerebrospinal fluid (CSF) and serum samples. Neves et al. [112] developed a disposable EC immunosensor for the simultaneous detection of IgA- and IgG-type anti-gliadin (GA) and anti-tissue transglutaminase (tTG) autoantibodies in real patient samples using dual screen-printed carbon electrodes as working electrodes. CD-specific antigens of GA and tTG were used as MREs, showing results that were consistent with commercial ELISA kits and allowing a decentralization of the analyses toward a POC strategy. 

Other techniques such as giant magnetoresistive (GMR) biosensor microarrays have been designed to analyze serum samples from SLE patients and have also been shown to be capable of identifying autoantibodies associated with relevant clinical manifestations of SLE, with potential for use as biomarkers in clinical practice [101].

Various biosensors for autoantibody detection used in recent years are listed in Table 2.

### 3.3. Evaluation of Vaccine Immunity Based on Biosensor

The follow-up evaluation of vaccine immunity is of great significance to estimate whether the contingent diseases could be effectively controlled. Besides monitoring infectious status and determining autoimmune disorders or allergies, antigen-based microarrays for antibody detection and characterization can also be used to evaluate the immunity efficiency of vaccines. As immunosensors for specific protection antibody detection are noninvasive, fast, highly accurate, and cheap, they can be used to develop new ways of evaluating clinical vaccine immunity. 

Similar to other immunological analysis in clinical labs, ELISA is the most common way to quantitatively determine protection antibodies. An array-based biosensor that simultaneously measures four different targets of toxins or viruses has been developed [125], showing an LOD as low as approximately 100 fg in human sera. The arrays can test 12 samples at once, providing a capacity for testing both positive samples and negative controls and for testing multiple serum samples and multiple dilutions. However, only a limited number of reported sensing platforms have been constructed for vaccine immunity evaluation. Arrays for antibody immunosensing evidently open new avenues for improving the efficiency of this.

## 4. Conclusions

Modern clinical laboratory diagnosis tends to develop in the directions of rapid analysis, high accuracy, easy operation, low cost, and miniaturization. Serological protein quantitative determination provides a novel tool giving fast results with high precision. Biosensors combined with various detection techniques have shown plenty of advantages for bioanalysis to further increase detection sensitivity, especially for serological immunoassays with low content. With the assistance of nanotechnology and diverse signal amplification strategies, the previously reported biosensors have shown attractive opportunities for protein detection. As mentioned above, biosensors for tumor markers, i.e., the typical protein targets in clinical immunology, make multi-channel testing possible, exhibiting high sensitivity and selectivity, easy operation, and low cost. Detection sensitivities of the reported biosensors show great potential for clinical applications, as we have described in Section 2. As the cutoff values of most antigens/antibodies can reach the ng mL^−1^ level, stability and easy operation for these devices, instead of higher sensitive detection strategies, should be pursued.

Antibody detection is widely needed for auxiliary diagnosis and has even become the golden standard for some infectious diseases, such as HIV. Very often, the primary biomarkers of diseases possess very low stability or are not present in body fluid at all. Thus they cannot be detected using the available detection techniques. Thus, specific antibodies have become the best biomarkers for clinical diagnosis. This method is very popular in the diagnosis of infectious, and other, diseases. We presented a brief summary of relevant biosensors for antibody detection with excellent performances in Section 3. These biosensing strategies will provide great opportunities for antibody detection in clinical serodiagnosis application. In addition, very few biosensors for vaccine-specific protection antibodies have been investigated, a prospective area of interest in serodiagnosis studies.

## Figures and Tables

**Table 1:** Biosensors constructed for biomarker detection of infectious diseases.

Disease	Biomarker	Biosensor Type	LOD	Reference
CCHF	Specific IgG antibodies	CL	ND	[3]
hepatitis B	hepatitis B surface antibodies	Surface acoustic wave	10 pg μL^−1^	[5]
	human IgG antibodies anti-HBsAg	Chronoampero-metric detection	3 mIU mL^−1^	[90]
HIV	anti-HIV antibody	ELISA	ND	[6]
Hepatitis C	anti-HCV antibodies	EC	0.003 pg mL^−1^	[33]
		CL	ND	[89]
Leishmaniasis	anti-leishmania infantum antibodies	PEC	0.05 mM	[55]
Malaria	anti-plasmodium vivax antibodies	EC	6 pg L^−1^	[82]
Cholera	anti-cholera toxin antibodies	EC	10^−13^ g mL^−1^	[83]
Neospora	anti-neospora antibodies	PL	ND	[85]
*Salmonella enteritidis* infection	egg yolk antibodies	SPR	ND	[86]
Adenoviruses infection	anti-adenoviruses antibodies	SPR	10 PFU mL^−1^	[87]
Dengue	IgM antibody	CL	ND	[88]

**Table 2:** Biosensors constructed for AID biomarker detection.

Disease	Biomarker	Biosensor Type	LOD	Ref.
APS	Multidetection of anti-β2-GPI antibody, anti-cardiolipin antibody, anti-phospholipid antibody, and anti-prothrombin antibody	Optical sensor	ND	[91]
	anti-β2-GPI antibody	Reflectometric interference spectroscopy	5.62 mg L^−1^	[92]
		SPR	ND	[93]
	Multidetection of anti-β2-GPI antibody, prothrombin, cardiolipin, and β2-GPI/cardiolipin complex	Reflectometric interference spectroscopy	ND	[94]
RA	anti-cyclic citrullinated peptide (CCP) autoantibody	EC	ND	[95]
		Surface-enhanced Raman scattering (SERS)	13 pg mL^−1^	[96]
		PL	0.1 ng mL^−1^	[97]
		piezoelectric	ND	[98]
		SPR	ND	[99]
SLE	Multidetection of anti-FLAG antibody, anti-Ro52 antibody, anti-U1-70K antibody, anti-K5Ac antibody, and anti-K20Ac antibody	Magneto resistive	ND	[101]
	anti-TRIM21 and anti-TROVE2 circulating autoantibodies	Piezoelectric	1.51 U mL^−1^ and 0.32 U mL^−1^	[102]
	anti-DNA autoantibody	EC	ND	[103]
		SPR	ND	[104]
		SPR	ND	[105]
		Piezoelectric	ND	[106]
	anti-chromatin autoantibody	EC	ND	[107]
MS	anti-glucopeptide antibody	SPR	ND	[108]
	anti-CSF114(Glc) antibodies	EC	ND	[109]
		SPR	ND	[110]
	anti-myelin basic protein (anti-MBP)	EC	0.1528 ng mL^−1^	[111]
CD	Multidetection of anti- GA and anti-tTG autoantibodies	EC	2.45 U mL^−1^ for tTG IgA and 2.95 U mL^−1^ for tTG IgG	[112]
	anti-GA autoantibodies	EC	0.52 arbitrary units mL^−1^	[113]
		EC	46 ng mL^−1^	[114]
		EC	9.1 U mL^−1^ for IgA and 9.0 U mL^−1^ for IgG	[115]
		Piezoelectric	100 nM	[116]
	anti-tTG autoantibodies	EC	260 ng mL^−1^	[117]
		EC	1.7 AU mL^−1^ for IgA and 2.7 AU mL^−1^ for IgG	[118]
		EC	ND	[119]
		EC	390 ng mL^−1^	[120]
		EC	20 A.U.	[121]
		EC	30 pM	[122]
		EC	ND	[123]
		Piezoelectric	1.3 μg mL^−1^	[124]

## Abbreviations

Ig	immunoglobulin
WHO	World Health Organization
IUIS	International Federation of Immunological Societies
AIDs	autoimmune diseases
APS	antiphospholipid syndrome
RA	rheumatoid arthritis
SLE	systemic lupus erythematosus
MS	multiple sclerosis
CD	celiac disease
ELISA	enzyme-linked immunosorbent assay
POC	point-of-care
MRE	molecular recognition element
EC	electrochemical
NPs	nanoparticles
CNTs	carbon nanotubes
CV	cyclic voltammograms
NRs	nanorods
LOD	limit of detection
SWCNTs	sing-walled carbon nanotubes
MWCNTs	multi-walled carbon nanotube
ITO	indium tin oxide
DPV	pulse voltammetry
SWV	square wave voltammetry
AFP	alpha fetoprotein
HRP	horseradish-peroxidase
PL	photoluminescent
HCV	hepatitis C virus
QDs	quantum dots
FRET	förster resonance energy transfer
cTnI	cardiac Troponin I
cTnT	cardiac troponin T
AFB1	aflatoxin B1
GVA-antigens	grapevine virus A-type proteins
ALD	atomic layer deposition
PEC	photoelectrochemical
g-C_3_N_4_	graphitic carbon nitride
CL	chemiluminescence
CEA	carcinoembryonic antigen
CA19-9	cancer antigen 19-9
ECL	electrochemiluminescent
PAMAM	polyamidoamine dendrimer
IFN-γ	interferon-gamma
TNF-α	tumor necrosis factor-alpha
IL-2	interleukin
FIA	fluidic immunoassay
CCHF	crimean-congo hemorrhagic fever
SPR	surface plasmon resonance
MCL	microcantilever
EIS	electrochemical impedance spectroscopy
ACPAs	anti-citrullinated peptide antibodies
GMR	giant magnetoresistive
CSF	human cerebrospinal fluid
GA	gliadin
tTG	tissue transglutaminase
